# It’s time to go …

**DOI:** 10.1007/s00062-022-01228-0

**Published:** 2022-12-12

**Authors:** László Solymosi

**Affiliations:** Dept. of Neuroradiology, University Hospital Würzburg, Würzburg, Germany

In 2005, I took on the role of Editor-in-Chief of *Clinical Neuroradiology*. From the first issue in 2006, I took care of the processes of this journal, founded in 1991, with the support of my younger colleagues as members of the editorial board. In these past 17 years, we have succeeded in turning a German language journal, which consequently had a limited readership only, into a widely read international scientific journal with a respectable Impact Factor.

Without your support as readers, reviewers, and authors of our journal, this would not have been possible. The current position of the journal among the scientific neuroradiological journals was only achieved because we received a high number of scientifically excellent submissions from you and because you continuously supported the journal with reviews of high quality, which enabled us to select the papers to be published in the journal.

I would like the journal to remain on the cutting edge scientifically. For a neuroradiologist who is no longer active in his profession and who no longer interacts with patients on a day to day basis, who therefore no longer receives professional “updates” and “upgrades” directly, this is not possible while maintaining the quality desired. For this reason, I am passing on the position of Editor-in-Chief at the end of this year. I am glad that we were able to find someone from the current editorial board as successor, who already has an in-depth experience with the journal. Martin Bendszus will take over the position of Editor-in-Chief on 1 January 2023.

As I bid farewell, I would like to thank the readership of *Clinical Neuroradiology,* the members of the editorial board, and also the publisher Springer Nature for their support over the past 17 years. Please continue to support *Clinical Neuroradiology* with excellent scientific papers. The editorial board still relies on your high-quality reviews, so please continue to support the journal with your expertise if at all possible. Support the journal, as it is the official journal of the German, Austrian and Swiss Societies for Neuroradiology and thus also a figurehead of German-speaking neuroradiology.

László Solymosi

Editor-in-Chief

